# Life-Threatening Obstetrical Emergency: Spontaneous Uterine Rupture Associated with Placenta Percreta in the First Trimester of Pregnancy—Case Report and Literature Review

**DOI:** 10.3390/reports7010021

**Published:** 2024-03-18

**Authors:** Mihaela Amza, Mihai-George Loghin, Didel-Ionuț Vâlcea, Nicolae Gică, Ileana-Maria Conea, Gabriel-Petre Gorecki, Alexandra Mirică, Romina-Marina Sima, Liana Pleș

**Affiliations:** 1 Department of Obstetrics and Gynecology, “Carol Davila” University of Medicine and Pharmacy, 020021 Bucharest, Romania; mihaela.amza@umfcd.ro (M.A.); mihai-george.loghin@drd.umfcd.ro (M.-G.L.); gica.nicolae@umfcd.ro (N.G.); ileana.conea@umfcd.ro (I.-M.C.); liana.ples@umfcd.ro (L.P.); 2 The “Bucur” Maternity, “Saint John” Hospital, 040294 Bucharest, Romania; ionutvalcea@gmail.com; 3 Filantropia Clinical Hospital Bucharest, 011132 Bucharest, Romania; 4 Faculty of Medicine, Titu Maiorescu University, 040441 Bucharest, Romania; gabriel.gorecki@prof.utm.ro; 5 Department of Anesthesia and Intensive Care, CF2 Clinical Hospital, 011464 Bucharest, Romania; 6 Department of Physiology I, “Carol Davila” University of Medicine and Pharmacy, 020021 Bucharest, Romania; alexandra.mirica@umfcd.ro

**Keywords:** placenta percreta, first trimester, placenta accreta spectrum, uterine rupture

## Abstract

Background: The greatest risk for the occurrence of the placenta accreta spectrum (PAS) is represented by uterine scars, which most frequently result after cesarean sections. Uterine rupture is a rare condition and appears mainly in the third trimester of pregnancy. The association between these two conditions is extremely rare in the first trimester of pregnancy. Methods: We performed a systematic review of abnormal placental adhesions and spontaneous uterine ruptures in the first trimester of pregnancy. We also reported a case of spontaneous uterine rupture in a 12-week pregnancy that presented with massive hemoperitoneum and hemorrhagic shock. Results: A 33-year-old patient with two previous cesarean sections, at the twelfth week of pregnancy at the time to this visit to the emergency room, presented with syncope and intense pelvic–abdominal pain. A clinical examination and ultrasound scan established the diagnosis of hemoperitoneum and hemorrhagic shock. Surgical exploration was performed, uterine rupture was identified, and hemostasis hysterectomy was necessary. The histopathological results showed placenta percreta. There have been eight reported cases of spontaneous uterine rupture in the first trimester of pregnancy associated with PAS. In these cases, it was found that 62.5% of the patients had undergone at least one cesarean section in the past; in 75% of the cases, hysterectomy was performed; and, in 87.5% of the cases, the presence of placenta percreta was confirmed. Conclusions: A high rate of cesarean sections determines the increase in the incidence of placenta accreta spectrum disorders. The possible life-threatening complications caused by this pathology can be observed in early pregnancies.

## 1. Introduction

The placenta accreta spectrum (PAS) refers to the trophoblastic tissue that abnormally invades the uterine wall. In 2019, the International Federation of Gynecology and Obstetrics (FIGO) published a classification for the diagnosis of PAS disorders. Grade 1 refers to an abnormally adherent placenta, also called placenta adherenta or creta. In this case, there are extensive areas where the placental tissue is directly attached to the superficial myometrium because there is no decidua between the myometrium and the placental villi. Grade 2 refers to placenta increta, which means a placenta with an abnormal invasion in the myometrium but without placental tissue being visualized invading the uterine serosa. Grade 3 is represented by placenta percreta, which has three subtypes which describe how the placenta invades the uterine serosa (grade 3a), the urinary bladder (grade 3b), or other pelvic organs (grade 3c) [1].

Several theories have been proposed to explain the appearance of PAS. The most widespread of them refers to a change in the endometrium–myometrium interface that does not allow the normal development of the trophoblastic tissue in the area of a uterine scar. The damage produced by a uterine scar can vary from a minimal lesion of the decidua and superficial muscle fibers to a deep and extensive defect of the myometrium. The decidua plays an important role in regulating trophoblastic infiltration. Trophoblastic tissue develops invasively in the scar area if there is no efficient re-epithelialization [2].

Placental invasion has been compared with carcinogenesis and the occurrence of metastases. It has been found that the process of epithelial-to-mesenchymal transition (EMT) is involved in both situations as well as a series of molecular biomarkers such as the vascular endothelial growth factor (VEGF), laminin γ2 (LAMC2), the placental growth factor (PlGF), E-cadherin (CDH1), transforming growth factor β (TGF-β), interleukin-35 (IL-35), zinc finger E-box-binding homeobox (ZEB) proteins, cofilin-1 (CFL-1), and αVβ3 integrin. Similarities have been found between these two processes regarding EMT promotion, invasion, angiogenesis, adhesion, and cell proliferation [3].

In 2019, Jauniaux et al. presented the results of a meta-analysis that showed that the overall pooled prevalence of PAS was 0.17% (ranged from 0.01 to 1.1%). This prevalence was increasing compared to the values published in the past, due to the increase in the incidence of the risk factors. The most frequent type of PAS disorders was the placenta accreta [4].

Over time, several risk factors for PAS have been identified, including placenta previa, a history of cesarean section or another uterine surgery, maternal age over 35 years [5], a pregnancy implanted on a cesarean scar [6], techniques of assisted human reproduction, especially cryopreserved embryo transfers [7], twin gestation [8], multiparity [9], smoking, and hypertensive disorders of pregnancy [10]. Uterine rupture is a risk factor for PAS disorders because it requires surgical intervention to restore the anatomical integrity of the uterus, and the presence of uterine scars is a risk factor for PAS disorders [11].

The following risk factors for uterine rupture have been identified: the presence of uterine scars, including a previous cesarean section, multiparity, uterine anomalies, tumors, fetal anomalies, PAS disorders, injudicious use of uterotonics, and cephalopelvic disproportion [12].

It is important that a PAS diagnosis be considered in the case of all patients with risk factors. The first evaluation method is represented by an ultrasound scan, during which specific signs of abnormal placental adhesion can be observed. In the first trimester of pregnancy, the following can be noticed: a gestational sac located in the lower uterine segment, numerous irregular vascular spaces in the placental tissue, and signs which suggest the implantation of the pregnancy on the C-section scar (cesarean section). Usually, MRI (magnetic resonance imaging) can confirm the diagnosis in the second or third trimester [13]. In a study by Abinader et al., it was found that the presence of at least three lacunae, the identification of Finberg’s grade 2 or 3 lacunae, the hypervascularization of the lower segment, and an abnormal uteroplacental interface were associated with the diagnosis of PAS in the first trimester of pregnancy [14].

A PAS disorder represents a potentially life-threatening pathology for both the mother and the fetus. PAS disorders can rarely cause uterine rupture, which can occur at any gestational age. In this case, the presence of abdominal pain, vaginal bleeding, or hemorrhagic shock can be noticed. It is important to establish the diagnosis as quickly as possible for the effective management of a case [15]. In rare cases, uterine rupture occurs before labor and the peripartum period [16].

## 2. Materials and Methods

In this paper, we present a case of hemoperitoneum with hemorrhagic shock in a patient at the twelfth week of pregnancy. The patient had undergone a single ultrasound scan at 7 weeks that confirmed the pregnancy, without specifying a C-section scar implantation. We hereby present the management of the patient and the intraoperative findings as well as the result of the pathological examination.

We also perform a systematic review of abnormal placental adhesions and spontaneous uterine ruptures in the first trimester of pregnancy. This analysis has been carried out in accordance with the recommendations made by Page et al. in 2020, indicated in “*The PRISMA 2020 statement: An updated guideline for reporting systematic reviews*” [17].

### 2.1. Literature Search

On the 6 August 2023, we searched the PubMed database using the following terms or keywords: “uterine rupture and first trimester”, “placenta percreta and uterine rupture”, “placenta accreta and uterine rupture”, “placenta increta and uterine rupture”, and “placenta percreta and first trimester”. We selected only case reports as a search filter, and we did not have any time or language restrictions for the publications.

### 2.2. Eligibility Criteria

We selected only the studies that simultaneously fulfilled the following inclusion criteria: (1) a gestational age under 14 weeks; (2) uterine rupture was caused by abnormal placental adhesion; (3) the result of the pathological examination was available; (4) uterine rupture was not iatrogenic (for example, as a result of dilation and curettage uterine or post-abortion medication); and (5) the full text of the article was available.

### 2.3. Selection of Studies and Data Collection

**Figure 1:**
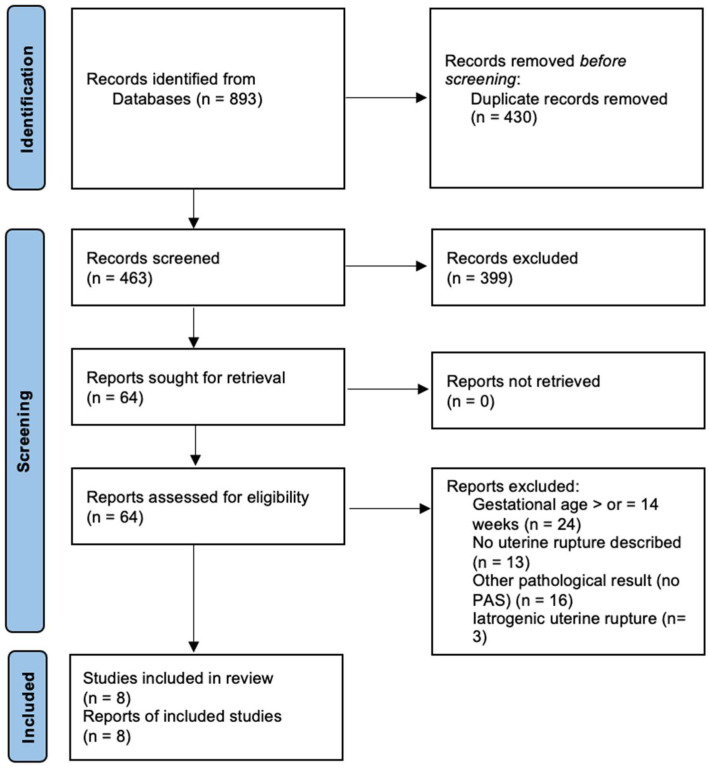
Flow chart.

The search in the database generated a total of 893 results. A number of 463 studies remained after the elimination of duplicates. This selection of studies was analyzed by two authors (R.-M.S and M.A.) under the supervision of L.P. The titles and abstracts were analyzed, and 64 articles were selected. After analyzing the full texts, only eight studies met all the inclusion criteria and were relevant for this analysis. The flow chart is presented below (Figure 1).

We collected the following data about each case: age of the patient, gestational age, presence of risk factors, previous C-section, symptoms, ultrasound scan, intraoperative findings, management, blood transfusion, and the result of the pathological examination.

### 2.4. Statistical Analysis

The collected data were used to create a database using the SPSS Statistic version 23 software (Armonk, NY, USA). Data analysis was performed using descriptive statistics, and the means, standard deviations, and frequencies were calculated.

## 3. Results

### 3.1. Case Report

A 33-year-old woman at the twelfth week of pregnancy presented herself to our emergency room for lower diffuse abdominal pain and syncope. She had undergone two cesarean sections in the past and had no other significant medical history. Her other two pregnancies had been uneventful. Until a few hours prior to this visit to the emergency room, this pregnancy had shown no special conditions. She had undergone an ultrasound scan that confirmed the pregnancy at 7 weeks, without mention of the abnormal C-section scar implantation of the gestational sac. This patient’s pregnancy care plan involved no other ultrasound scans even if the patient had an increased risk of developing PAS considering her history of two prior cesarean sections. This patient’s following evaluation had been scheduled for their thirteenth week of pregnancy, for the first trimester fetal morphology scan.

**Figure 2:**
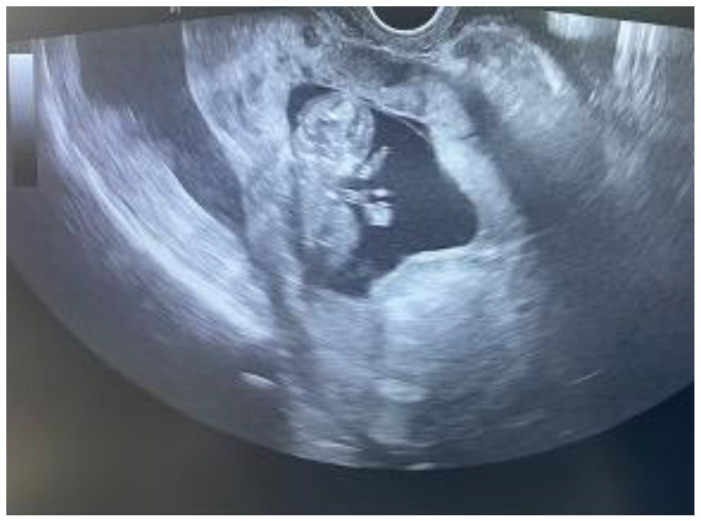
Transvaginal ultrasound scan.

**Figure 3:**
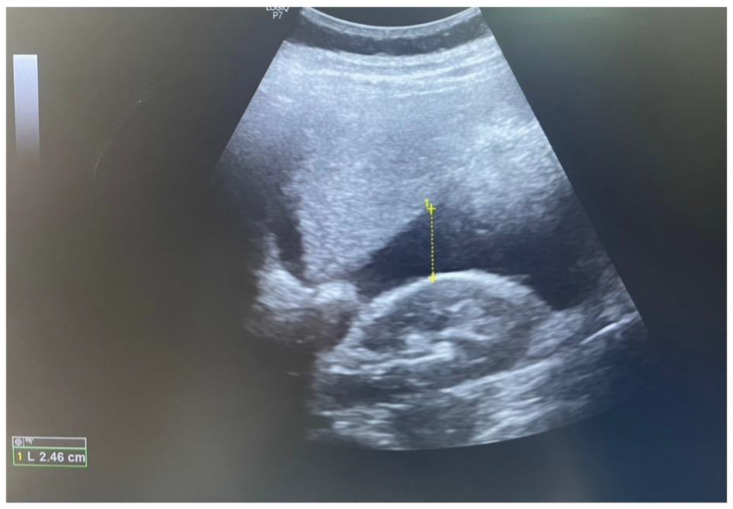
Abdominal ultrasound scan (yellow dotted line indicated the presence of fluid in the hepato-renal space).

At the time of admission, the clinical evaluation found the patient confused, uncooperative, and hypotensive (BP: 90/50 mmHg; HR: 113 bpm). The physical examination revealed paleness, signs of peritoneal irritation, and no vaginal bleeding. In the emergency room, we performed a transvaginal and abdominal ultrasound scan. The transvaginal ultrasound scan identified an intrauterine gestational sac with a single embryo with cardiac activity and an adequate size for 12 weeks of pregnancy (Figure 2). The cervix, with a normal length, was closed, and the internal ostium was completely covered by the lower segment of the placental tissue that ascended on the anterior uterine wall. At the C-section scar level, no myometrium or serosa could be visualized. An inhomogeneous area suggestive of blood accumulation was observed in the utero–vesical space. Fluid was present in the hepato-renal space and the retro-uterine space during the abdominal ultrasound scan (Figure 3).

**Figure 4:**
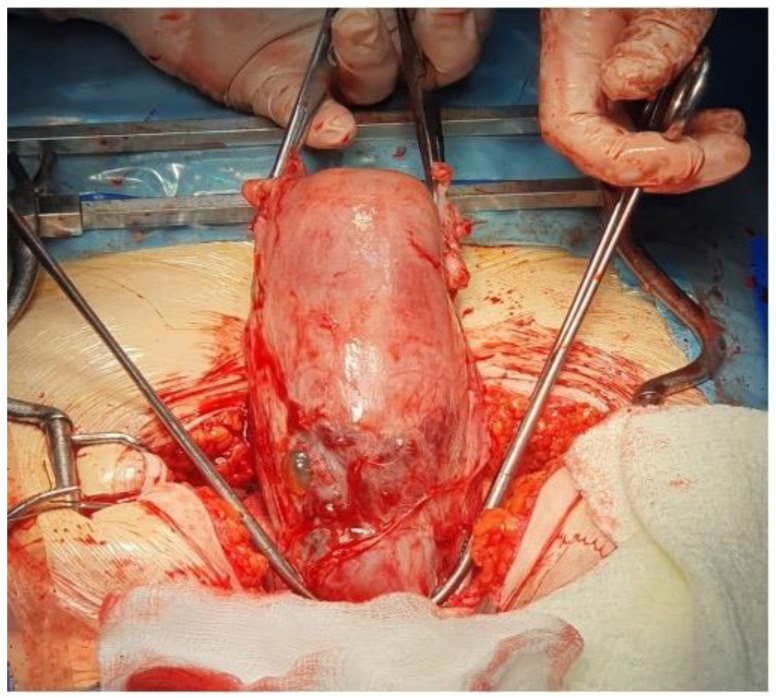
The uterine rupture.

We established a diagnosis of hemoperitoneum and hemorrhagic shock at 12 weeks of gestation (BP: 70/20 mmHg; HR 138 bpm). We decided to perform an emergency laparotomy. After opening the peritoneal cavity, we observed a massive hemoperitoneum, approximately 2000 mL of blood and clots. The pregnant uterus was identified. Active bleeding and ruptured vessels were observed in the C-section scar area. Placental tissue externalized through the uterine rupture at the same level was visualized (Figure 4). The length of the laceration area was approximately 2 cm. Considering the hemodynamic status of the patient, the huge bleeding potential, and the unavailability of other methods to ensure hemostasis (for example, uterine artery embolization), we decided to perform a hysterectomy. We also decided to perform a hysterectomy because there was an extensive area with macroscopic placental invasion, the patient had undergone two previous cesarean sections that had affected the quality and thickness of the uterine wall, and the urinary bladder was adherent to the anterior uterine wall. We considered that, after the excision of the myometrial area affected by placental invasion, restoring the anatomical integrity of the uterus would not have been possible. The first results of the blood tests became available during the surgery: hemoglobin 6.7 g/dL and hematocrit 19.8%. It was necessary to restore the patient’s hematological status during the surgery by transfusing two units of blood. The hysterectomy was performed safely and without incidents. No technical issues or complications were reported during the surgical procedure.

**Figure 5:**
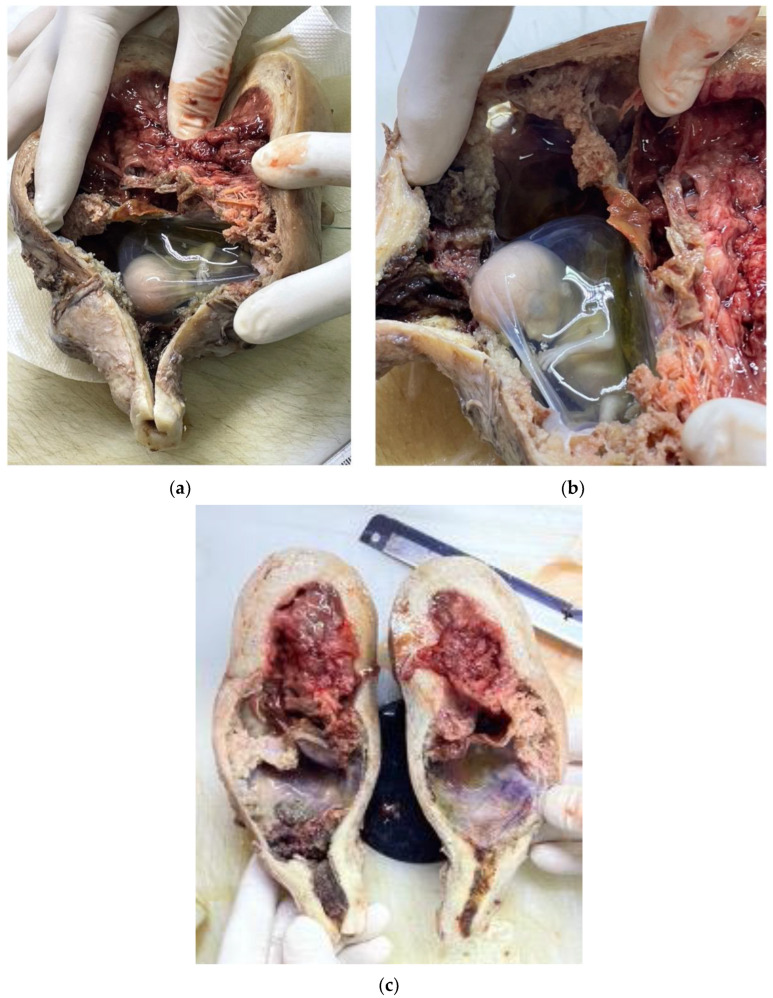
Pathological examination: sectioning of the uterus (**a**); uterine rupture (**b**); the lower location of the gestational sac and the placenta with thinning of the myometrium in this area (**c**).

**Figure 6:**
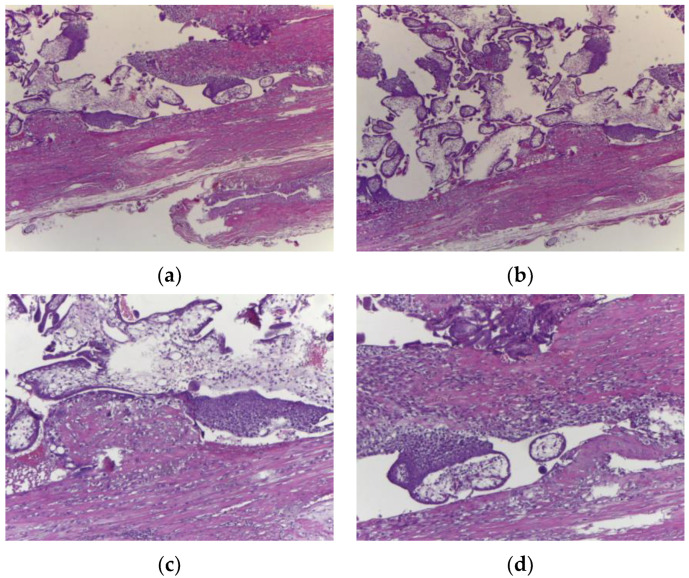
Microscopic examination: chorionic villi that invade the muscular mucosa, inserted up to the serosa (**a**,**b**); magnified images for details (**c**,**d**).

Images from the pathological examination are available (Figure 5). A microscopic examination confirmed the presence of a placenta with abnormal adhesion (Figure 6). A diagnosis of placenta percreta associated with uterine rupture at the C-section scar level was established. This was described as the rupture of the uterine wall also having affected the vessels on its surface.

The follow-up of the patient was favorable after surgery under anticoagulant, analgesic, and antibiotic treatments. In the postoperative period, the transfusion of one unit of plasma was necessary. The patient was discharged after five days. The patient returned for an evaluation after one week, and she had normal clinical parameters.

### 3.2. Results of Systematic Review

In this review, eight studies reporting eight cases of spontaneous uterine rupture in the first trimester of pregnancy due to PAS were included. The characteristics of the cases are summarized in Table 1. The first case was reported in 1999 by Marcus et al. [18]. In this case, spontaneous uterine rupture occurred at a gestational age varying between 7 and 13 weeks. The gestational age was 12 weeks in three cases (37.5%) and 13 weeks in another three cases (37.5%). The mother’s age varied between 22 and 40 years, with an average of 31.71 years and a standard deviation of 4.63 years. Out of the total of eight cases, only one patient was in her first pregnancy and got pregnant because of a frozen embryo transfer; all the other patients had at least given birth once before. A number of five (62.5%) patients presented a medical history with a minimum of one cesarean section. In the case of one patient, no risk factors were identified, and she had experienced two previous vaginal births. In the case presented by Esmas et al. [19], the patient had a history of vaginal birth but with a manual extraction of the placenta followed by a curettage for an abnormally adherent placenta that turned out to be accreta. In six out of the eight cases (75%), the management of the condition consisted of a hysterectomy. In the case presented by Marcus et al. [18], uterine artery embolization was performed, and methotrexate was administered. In a single case, presented by deRoux et al. [20], the diagnosis was established during an autopsy as the patient died 10 h after being admitted to the hospital, despite an attempt to restore their hemodynamics. In seven out of the eight cases (87.5%), the pathological examination confirmed the presence of a placenta percreta, and, in only one case (12.5%), it was a placenta accreta.

The data regarding the clinical evaluation of the patients are summarized in Table 2. In seven out of the eight cases (87.5%), abdominal pain of variable intensities was present, from mild to severe, and sudden abdominal pain. Other reported symptoms were vaginal bleeding, syncope, vomiting, and abdominal distension. Ultrasound scanning was reported in seven out of the eight cases (87.5%). In five out of the seven cases (71.42%), a presence of free liquid in the abdominal cavity was described. In four cases (57.14%), the presence of an intrauterine pregnancy with cardiac activity was described. Marcus et al. [18] described the presence of an abnormal localized pregnancy, while Hanif et al. [22] described the ultrasound scan as showing an ectopic pregnancy. Regarding the site of uterine rupture, it was found that, in three cases (37.5%), the uterine rupture was at the uterine fundus; in four cases (50%), the rupture was at the C-section scar level and in the lower segment; and, in one case, rupture of the right uterine horn was described. In two cases (25%), invasion of the urinary bladder by the placental tissue was also noticed. The necessity of blood transfusions was reported in half of the cases.

## 4. Discussion

We presented a case of hemoperitoneum and hemorrhagic shock in a patient at the twelfth week of her pregnancy. An emergency laparotomy was performed, and the presence of uterine rupture was found. A hysterectomy for hemostasis was necessary. The pathological result proved the presence of placenta percreta. We found another eight cases of spontaneous uterine rupture in the first trimester of pregnancy that were associated with abnormal placental adhesions. A history of C-sections is the main risk factor for the occurrence of PAS and was also present in our case.

In the literature, we found four other reported cases of uterine rupture associated with PAS at a gestational age of 14 weeks at the limit for inclusion in this study. The risk factors identified in these cases were a history of C-sections [26,27], gravid rudimentary horn on a bicornuate uterus [28], and pregnancy obtained through in vitro fertilization in a woman with Turner’s syndrome [29]. Except for the bicornuate uterus case, in which the rudimentary horn was resected, in the other cases, hysterectomy was performed.

There have been described cases with massive hemorrhages following uterine curettage in the first trimester in which laparotomy was performed and uterine rupture associated with PAS was found. Woolcott et al. described such a case at the tenth week of pregnancy [30], and Tanyi et al. described a similar case at 7 weeks of pregnancy [31]. Both patients had a history of C-sections.

The diagnosis of PAS disorders is difficult in the first trimester. A careful ultrasound scan is necessary in the case of patients who present risk factors. A systematic review by Guzmán López et al. showed that 74.54% of PAS disorder cases encountered in the first trimester are diagnosed during hospitalization for abortion. The most common symptoms were vaginal bleeding, abdominal pain, syncope, and nausea. In 22.49% of the cases included in the study by Guzmán López et al., they presented ultrasonographic signs of a C-section scar-implanted pregnancy. It is important that, in half of the cases, the diagnosis of PAS was achieved only by histological evaluation and that only in 21.15% of the cases the diagnosis was defined using ultrasound techniques [32].

Ultrasound scanning has a sensitivity of 41% and a specificity of 88% in the detection of placenta accreta in the first trimester of pregnancy. This sensitivity is lower than in the other trimesters, as it can be up to 60% in the second trimester and up to 83.5% in the third trimester. The most common signs of PAS disorders in the first trimester are represented by an irregular placental–myometrial interface, a low-lying gestational sac, placenta previa, and hypo echoic placental regions [33]. In the case of a hemoperitoneum, the gestational sac, an embryo with cardiac activity, and a fundic placenta can create confusion and a false impression that the hemorrhage is not of obstetric origin, which will delay the diagnosis and its correct management [21]. Most of the time, an MRI examination is the one that clarifies the diagnosis of PAS and is frequently performed in the third trimester of pregnancy [34].

In early pregnancies in which no risk factors can be identified, the diagnosis of uterine rupture is established with a delay, and, thus, life-threatening complications can appear that can lead to the death of the patient, as happened in the case reported by de Roux et al. [16]. Perdue et al. reported, in a review which included 61 patients with first-trimester pregnancies, that the diagnosis of uterine rupture was made by means of surgical exploration in 61% of the cases. They mentioned that a hysterectomy is not mandatory but that surgery is typically useful for establishing a diagnosis of uterine rupture. The management of the condition varied: defect repair was possible in 61% of the cases, and the pregnancies continued in four cases (7%) [35].

Uterine rupture is a rare pathology and is found especially in full-term pregnancies. Cecchini et al. carried out a systematic review that evaluated the cases of uterine rupture reported in the first trimester of pregnancy. A total of 76 cases were found. A previous uterine surgery was found in 69.74% of the cases, which included C-sections, myomectomies, dilation, and curettage or cornual resections. Other risk factors were represented by assisted human reproduction techniques in 11.84% of the cases, drug usage in 11.84% of the cases, and uterine anomalies (bicorn uterus, rudimentary horn, T-shaped uterus) in 15.79% of the cases. The most frequent localizations of the uterine rupture were at the C-section scar level or at the uterine fundus. The management of the condition was represented by a hysterectomy in 30.26% of the cases and by repairing the defect in 63.16% [36].

Vaezi et al. presented a review of eight cases of uterine rupture in an unscarred uterus at a gestational age below 20 weeks. It was found that, in all the cases, the rupture was located at the uterine fundus. In seven out of the eight cases (88%), multiparity represented a risk factor. Placenta percreta was observed only in two cases. The defect was repaired in half of the cases [37].

Most of the time, a series of risk factors are present, and the cause can be identified, but there are also cases in which these cannot be clearly specified. Park et al. reported a case of spontaneous uterine rupture at 10 weeks in which no risk factors were identified and the cause could not be mentioned [38].

Uterine rupture must be part of a differential diagnosis if a pregnant woman with a history of uterine surgery presents in the first trimester with severe and sudden-onset abdominal pain and also presents unstable vital signs. The risk of uterine rupture is higher if the patient has several associated medical or surgical conditions [39].

## 5. Conclusions

The high rate of cesarean sections has increased the incidence of the placenta accreta spectrum. The possible life-threatening complications caused by this disorder begin to be observed in the early stages of pregnancy. It is necessary to carefully evaluate patients with a previous cesarean section because they are the main candidates for PAS. It is important for physicians to be aware of this condition and not underestimate it. The early diagnosis of PAS disorders is useful in establishing their optimal management in these pregnancies and reducing maternal morbidity. Knowing the life-threatening complications of PAS that appear starting from the first trimester of pregnancy will be a trigger for the early diagnosis of these conditions.

## Figures and Tables

**Table 1:** The characteristics of the cases.

Author	Gestational Age (Weeks)	Maternal Age (Years)	Gesta Para	Risk Factors	Management	Type of PAS
Marcus et al. (1999) [18]	13	38	G4 P2	2 C-sections	Uterine arteries’ embolization and MTX before hysterectomy	percreta
deRoux et al. (1999) [20]	12	22	G9 P2	no risk factors	No surgery; maternal death	percreta
Esmas et al. (2004) [19]	13	40	G2 P1	history of placenta accreta	Hysterectomy	percreta
Dabulis et al. (2007) [21]	9	*	G5 P3	3 C-sections	Hysterectomy	percreta
Hanif et al. (2011) [22]	12	24	G5 P2	2 C-sections	Hysterectomy	percreta
Brown et al. (2015) [23]	12	28	G2 P1	1 C-section	Hysterectomy	percreta
Cho et al. (2017) [24]	7	34	G1 P0	frozen embryo transfer	Hysterectomy	percreta
Ambrogi et al. (2018) [25]	13	36	G2 P1	1 C-section	Excision of the edges of the uterine rupture and uterine reconstruction	Accreta
Our case	12	33	G3 P2	2 C-sections	Hysterectomy	Percreta

MTX: methotrexate; PAS: placenta accreta spectrum; and * no data available.

**Table 2:** Clinical findings.

Author	Clinical Presentation	Ultrasound Scan	Intraoperative Findings	Blood Transfusion
Marcus et al. (1999) [18]	cramping and spotting	Pregnancy with cardiac activity abnormally located (a saccular structure anterior but contiguous with the uterus); the endometrium was seen superior to but not surrounding the saccular structure	lower segment UR	*
deRoux et al. (1999) [20]	abdominal pain and unresponsive patient	Fetal death; no free fluid intraabdominally	fundal UR	*
Esmas et al. (2004) [19]	abdominal pain	Intrauterine pregnancy with cardiac activity; free fluid in the abdomen	fundal UR	yes
Dabulis et al. (2007) [21]	abdominal pain, bloating, vomiting, and diarrhea	Intrauterine pregnancy with cardiac activity; free fluid in the cul-de-sac	C-section scar UR	*
Hanif et al. (2011) [22]	vomiting, syncope, sharp lower abdominal pain, and abdominal distension	Extrauterine pregnancy; moderate free fluid in the peritoneal cavity	C-section scar UR; bladder invasion	*
Brown et al. (2015) [23]	syncopal episode and vaginal bleeding	*	lower segment UR; bladder invasion	yes
Cho et al. (2017) [24]	sudden severe abdominal pain	Intrauterine pregnancy with cardiac activity; free fluid in the abdominal cavity	fundal UR	*
Ambrogi et al. (2018) [25]	moderate abdominal pain	Intrauterine pregnancy with cardiac activity; free fluid next to the liver and the spleen and in the pelvis	right uterine horn rupture	*
Our case	lower diffuse abdominal pain and syncope	Intrauterine pregnancy with cardiac activity; free fluid next to the liver and in the pelvis; placental tissue on the anterior uterine wall; no myometrium or serosa at the level of the C-section scar	C-section scar UR	yes

UR: uterine rupture; and * no data available.

## Data Availability

The data used in this study are available from the corresponding author, and the authors can share the information if there is a reasonable request.
